# Supplementary material for: Multiplex PCR Pneumonia Panel in Critically Ill Patients Did Not Modify Mortality: A Cohort Study
Source: Antibiotics (Basel). 2025 Feb 28;14(3):245. doi: 10.3390/antibiotics14030245 (PMC11939521; doi:10.3390/antibiotics14030245)
Supplement: Supplementary file 1 [file antibiotics-14-00245-s001.zip › antibiotics-3431296-supplementary.pdf]

Supplementary material:

Table S1
Microorganisms isolated in respiratory sample cultures

	Microorganism	Total n=153	PN-panel n=105	No PN-panel n=48
**Gram Negative Rods**	*Klebsiella pneumoniae*	32 (20.9)	19 (18.1)	13 (27.1)
	Escherichia coli	14 (9.2)	13 (12.4)	1 (2.1)
	Haemophilus influenzae	9 (5.9)	6 (5.7)	3 (6.3)
	Enterobacter cloacae	8 (5.2)	3 (2.9)	5 (10.4)
	Serratia marcescens	6 (3.9)	5 (4.8)	1 (2.1)
	Proteus mirabilis	3 (2.0)	2 (1.9)	1 (2.1)
	Haemophilus parahaemolyticus	3 (2.0)	3 (2.9)	0 (0)
	Klebsiella aerogenes	3 (2.0)	2 (1.9)	1 (2.1)
	Klebsiella oxytoca	2 (1.3)	0 (0)	2 (4.2)
	Citrobacter koseri	2 (1.3)	1 (1.0)	1 (2.1)
	Haemophilus parainfluenzae	2 (1.3)	1 (1.0)	1 (2.1)
	Citrobacter freundii	1 (0.7)	1 (1.0)	0 (0)
	Klebsiella spp	1 (0.7)	1 (1.0)	0 (0)
**Gram Positive Cocci**	*Staphylococcus aureus*	21 (13.7)	15 (14.3)	6 (12.5)
	Enterococcus faecalis	2 (1.3)	2 (1.9)	0 (0)
	Streptococcus oralis	1 (0.7)	1 (1.0)	0 (0)
	Streptococcus sanguinis	1 (0.7)	1 (1.0)	0 (0)
**Fungi**	*Candida albicans*	23 (15.0)	15 (14.3)	8 (16.7)
	Candida tropicalis	2 (1.3)	2 (1.9)	0 (0)
	Candida glabrata	1 (0.7)	0 (0)	1 (2.1)
	Aspergillus flavus complex	1 (0.7)	0 (0)	1 (2.1)
**Non Fermentative Gram Negative**	*Pseudomonas aeruginosa*	5 (3.3)	2 (1.9)	3 (6.3)
	Acinetobacter pittii	2 (1.3)	2 (1.9)	0 (0)
	Burkholderia cepacia	1 (0.7)	0 (0)	1 (2.1)
	Stenotrophomonas maltophilia	1 (0.7)	1 (1.0)	0 (0)
**Gram Negative Cocci**	*Moraxella catarrhalis*	1 (0.7)	1 (1.0)	0 (0)
**Others**	Negative	23 (15.0)	18 (17.1)	5 (10.4)
	Polymicrobian	16 (10.5)	12 (11.4)	4 (8.3)

Table S2 Microorganisms isolated in blood cultures

	Microorganisms	Total n=211	PN-Panel n=128	No PN-Panel n=83
**Gram Negative Rods**	*Klebsiella pneumoniae*	10 (4.7)	4 (3.1)	6 (7.2)
	Escherichia coli	5 (2.4)	2 (1.5)	3 (3.6)
	Serratia marcescens	5 (2.4)	4 (3.1)	1 (1.2)
	Haemophilus influenzae	1 (0.5)	1 (0.8)	0 (0)
	Enterobacter cloacae	1 (0.5)	1 (0.8)	0 (0)
	Proteus mirabilis	1 (0.5)	0 (0)	1 (1.2)
	Klebsiella oxytoca	1 (0.5)	1 (0.8)	0 (0)
	Salmonella enterica	1 (0.5)	0 (0)	1 (1.2)
**Gram Positive Cocci**	*Staphylococci coagulase negative*	21 (10.0)	15 (11.7)	6 (11.6)
	Staphylococcus aureus	6 (2.8)	3 (2.3)	3 (3.6)
	Streptococcus mitis/oralis	2 (0.9)	2 (1.5)	0 (0)
	Streptococcus agalactiae	2 (0.9)	1 (0.8)	1 (1.2)
	Streptococcus pneumoniae	1 (0.5)	0 (0)	1 (1.2)
	Enterococcus faecalis	1 (0.5)	0 (0)	1 (1.2)
	Streptococcus vestibularis	1 (0.5)	1 (0.8)	0 (0)
**Fungi**	*Candida albicans*	1 (0.5)	0 (0)	1 (1.2)
**Non fermentative Gram Negative**	*Pseudomonas aeruginosa*	3 (1.4)	1 (0.8)	2 (2.4)
	Burkholderia cepacia	1 (0.5)	0 (0)	1 (1.2)
	Stenotrophomonas maltophilia	2 (0.9)	2 (1.5)	0 (0)
**Others**	Negative	155 (73.4)	96 (75.0)	59 (71.1)

Table S3 PN-panel detection targets

PN-panel detection targets	Total n=150 n(%)
**Bacteria**	
Bacteria detected	90 (60.0)
Mixed detection	40 (44.4)
Acinetobacter calcoaceticus-baumannii complex	5 (3.3)
Enterobacter cloacae complex	9 (6.0)
Escherichia coli	26 (17.3)
Haemophilus influenzae	18 (12.0)
Klebsiella aerogenes	2 (1.3)
Klebsiella oxytoca	1 (0.7)
Klebsiella pneumoniae group	29 (19.3)
Moraxella catarrhalis	5 (3.3)
Proteus sp	4 (2.7)
Pseudomonas aeruginosa	6 (4.0)
Serratia marcescens	5 (3.3)
Staphylococcus aureus	39 (26.0)
Streptococcus agalactiae	12 (8.0)
Streptococcus pneumoniae	8 (5.3)
Streptococcus pyogenes	2 (1.3)
Legionella pneumophila	0 (0)
Mycoplasma pneumoniae	0 (0)
Chlamydia pneumoniae	0 (0)
**Resistance Genes**	
Resistance gen detected	29 (19.3)
ESBL	0 (0)
CTX-M	10 (6.7)
KPC	11 (7.3)
NDM	2 (1.3)
Oxa-48 like	2 (1.3)
VIM	2 (1.3)
IMP	0 (0)
mecA/mecC y MREJ	16 (10.7)
**Virus**	**%**
Virus detected	20 (13.3)
Influenza A	3 (2.0)
Influenza B	1 (0.7)
Adenovirus	2 (1.3)
Coronavirus	2 (1.3)

Parainfluenza	2 (1.3)
Respiratory Syncytial Virus	1 (0.7)
Rhinovirus/enterovirus	11 (7.3)
Human Metapneumovirus	0 (0)
MERS-CoV	0 (0)

Table S4 Concordance between isolation on respiratory sample cultures and detections of PN-panel.

**Respiratory sample cultures isolations n=105**						
	**RC+ PN+panel+**	**RC+ PN-panel-**	**RC- PN-panel+**	**RC- PN-panel-**	**Kappa**	**Concordance**
Acinetobacter calcoaceticus/baumannii	2	0	3	100	0.56	97%
Enterobacter cloacae	1	2	4	98	0.22	94.3%
Klebsiella aerogenes	2	0	0	103	1	100%
Escherichia coli	12	1	7	85	0.71	92.4%
Haemophilus influenzae	4	2	10	89	0.35	88.6%
Klebsiella oxytoca	0	0	1	104	0	99%
Klebsiella pneumoniae	14	6	7	78	0.61	87.6%
Moraxella catarrhalis	1	0	4	100	0.32	96.2%
Proteus spp	1	1	2	101	0.39	97.1%
Pseudomonas aeruginosa	1	1	3	100	0.32	96.2%
Serratia marcescens	2	3	2	98	0.42	95.2%
Staphylococcus aureus	13	2	14	76	0.53	84.8%
Streptococcus agalactiae	0	0	7	98	0	93.3%
Streptococcus pneumoniae	0	0	8	97	0	92.4%
Streptococcus pyogenes	0	0	1	104	0	99%

RC: Respiratory sample cultures, PN-panel: multiplex PCR pneumonia panel

Table S5 Concordance between respiratory sample cultures CFU and copies/ml of PN-Panel

	Respiratory Sample Culture (CFU)			
**PNB-Panel / (copies/ml)**	Negative	10^3	10^4	$\geq 10^5$
Non detected	1434	1	3	6
10^4	6	0	1	3
10^5	0	0	7	5
10^6	2	0	3	6
10^7	3	1 *	6	21
Without data	0	0	0	1
Agreement (%)	99.2 (1434/1445)	50 (1/2)	5 (1/20)	76.2 (32/42)

* Respiratory sample culture reported as *Haemophilus influenzae* poor growth”
CFU: Colony Forming Units, PN-Panel (Multiples PCR Pneumia Panel)

Table S6
Definitions of Infectious Diseases Decision

Decision	Definition
**None**	If the patient was evaluated by the infectious disease team, it was defined as none if no decisions were made in the note regarding the antimicrobial regimen initiated.<br>If the patient was not evaluated by infectious diseases, the behavior was also marked as none.
**Endorsement**	If the patient was evaluated by infectious diseases team, it was defined as an endorsement if the medical history record approved both the antimicrobial regimen, and the dose initiated.
**Adjustment**	If the patient was evaluated by infectious diseases team, it was defined as an adjustment if in the medical history record there was evidence of changes in dosage, changes in any of the components of the scheme and not in all of them, or a change in the objective of the antimicrobial coverage.
**Increase spectrum**	If the patient was evaluated by infectious diseases team, it was defined as escalating if the decision to increase the spectrum of antimicrobial coverage was recorded in the medical history.
**Decrease spectrum**	If the patient was evaluated by infectious diseases team, it was defined as de-escalation if there was a record of the decision to decrease the spectrum of antimicrobial coverage in the medical history record.
**Suspension**	If the patient was evaluated by infectious diseases team, it was defined as discontinuation if there was a record of the decision to completely discontinue antimicrobials in the medical history record.

Figure S1

SMD of de original population and post IPTW pseudopopulation

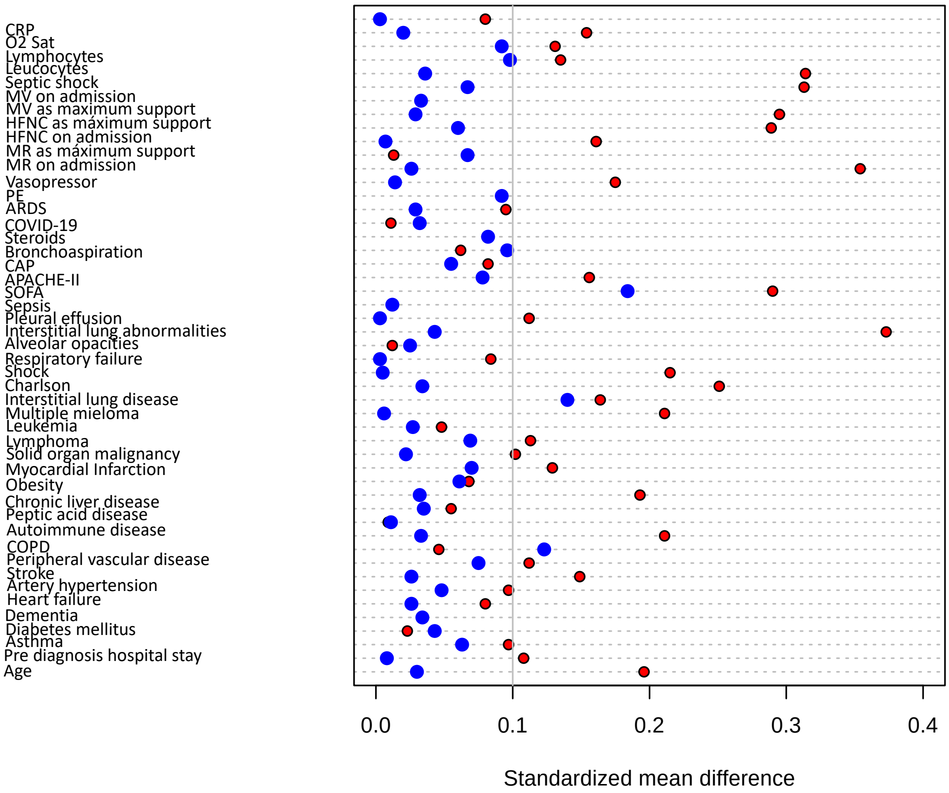

Inverse probability of treatment weighting (IPTW), CRP (C reactive Protein) SMD (standardized mean differences), O2 Sat (Oxygen Saturation), MV (Mechanical Ventilation), HFNC (High Flow Nasal Canula), MR (Mask with Reservoir), PE (Pulmonary Embolism), ARDS (Acute Respiratory Distress Syndrome), CAP (Community Acquired Pneumonia), APACHE-II (Acute Physiology, Age, and Chronic Health Evaluation Score), SOFA (Sequential Organ Failure Assessment), COPD (Chronic Obstructive Pulmonary Disease).
